# Paraneoplastic Pemphigus: Paraneoplastic Autoimmune Disease of the Skin and Mucosa

**DOI:** 10.3389/fimmu.2019.01259

**Published:** 2019-06-04

**Authors:** Jong Hoon Kim, Soo-Chan Kim

**Affiliations:** Department of Dermatology and Cutaneous Biology Research Institute, Gangnam Severance Hospital, Yonsei University College of Medicine, Seoul, South Korea

**Keywords:** paraneoplastic pemphigus, neoplasms, tolerance, humoral immunity, cell-mediated immunity

## Abstract

Paraneoplastic pemphigus (PNP) is a rare but life-threatening mucocutaneous disease mediated by paraneoplastic autoimmunity. Various neoplasms are associated with PNP. Intractable stomatitis and polymorphous cutaneous eruptions, including blisters and lichenoid dermatitis, are characteristic clinical features caused by humoral and cell-mediated autoimmune reactions. Autoreactive T cells and IgG autoantibodies against heterogeneous antigens, including plakin family proteins and desmosomal cadherins, contribute to the pathogenesis of PNP. Several mechanisms of autoimmunity may be at play in this disease on the type of neoplasm present. Diagnosis can be made based on clinical and histopathological features, the presence of anti-plakin autoantibodies, and underlying neoplasms. Immunosuppressive agents and biologics including rituximab have been used for the treatment of PNP; however, the prognosis is poor due to underlying malignancies, severe infections during immunosuppressive treatment, and bronchiolitis obliterans mediated by autoimmunity. In this review, we overview the characteristics of PNP and focus on the immunopathology and the potential pathomechanisms of this disease.

## Introduction

Paraneoplastic pemphigus (PNP) is a rare mucocutaneous autoimmune disease associated with neoplasm (1). Since Anhalt et al. (1) first proposed diagnostic criteria for PNP in 1990, revised criteria have been proposed by several research groups (2–5). Although consensus guidelines have not been reached, four features are consistently found in the majority of PNP patients and are generally accepted with a high degree of confidence as the minimal criteria for diagnosis. These features include (1) clinical features of severe and persistent stomatitis with or without polymorphic cutaneous eruptions, (2) histologic features of acantholysis and/or interface dermatitis, (3) demonstration of anti-plakin autoantibodies, and (4) presence of an underlying neoplasm. PNP manifests as polymorphic mucocutaneous eruptions mediated by humoral and cellular immunity. Moreover, the autoimmune reaction can appear in internal organs, such as the lung. Considering this potential lung involvement, the more inclusive term, “paraneoplastic autoimmune multi-organ syndrome,” has been proposed for this disease (6). Less than 500 cases of PNP have been reported worldwide in patients with various clinical features and autoantibody profiles (7). PNP is genetically associated with the human leukocyte antigen (HLA)-Cw^*^14 and HLA-DRB1^*^03 (8, 9). Tumors associated with PNP are mostly hematologic malignancies, including lymphoma, leukemia, and Castleman disease (10, 11). The mortality rate is high because of severe infections (e.g., sepsis and pneumonia), underlying malignancy, or bronchiolitis obliterans which is related to the autoimmune response.

## Disease Manifestations

### Clinical Features

**Figure 1:**
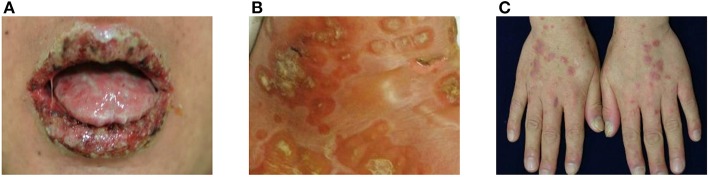
Clinical manifestations of paraneoplastic pemphigus (PNP). **(A)** Extensive erosions with ulcers and crusts are shown on the vermilion borders of the lips. **(B)** Blisters and erythematous patches with crusts are observed. **(C)** Erythematous to violaceous papules and plaques with silvery scales are present on the dorsum of hands.

The most characteristic feature of PNP is stomatitis, which usually is the first presenting sign and persists over the course of the disease (2, 12). Stomatitis presents as erosions and ulcerations affecting the oropharynx and extending to the vermilion border of the lips (Figure 1A). In addition to stomatitis, mucositis involving the pharynx, larynx, and esophagus can occur (2). Moreover, conjunctivitis is also common in these patients, sometimes causing visual impairment (13), and anogenital involvement is also observed in PNP (14). In several cases, mucosal involvement is the only sign of PNP (15–17).

Skin lesions of PNP are polymorphic and may appear with different features in the same patient. Blisters and erosions are commonly observed and mimic those of pemphigus vulgaris, pemphigus foliaceus, or bullous pemphigoid, affecting any area of the body (Figure 1B). The blisters may be confluent, similar to that in toxic epidermal necrolysis, or may be erythema multiforme-like targetoid lesions. Another type of characteristic cutaneous lesions are lichenoid eruptions, which manifest as erythematous papules and plaques, similar to that in lichen planus and graft-vs.-host disease (Figure 1C). In some cases of PNP, cutaneous lesions may present as onychodystrophy and alopecia (14). As for extracutaneous lesions, bronchiolitis obliterans, one of the major causes of death in PNP, is found in ~30% of PNP patients and frequently develops in patients with Castleman disease (18, 19). The initial symptom of bronchiolitis obliterans is dyspnea, and pulmonary function tests show obstructive lung disease (2).

### Associated Neoplasms

PNP is associated with underlying neoplasms, the most frequent of which are hematologic malignancies. Previous studies revealed that non-Hodgkin lymphoma (about 40%) is the most frequent neoplasm, followed by Castleman disease (15~37%) and chronic lymphocytic leukemia (CLL) (7~18%) (10, 11, 20). Castleman disease has been reported as the most frequent neoplasm in Korea and China (21, 22), suggesting that the incidence of associated neoplasms vary by ethnicity. Castleman disease is the most commonly associated neoplasm in children with PNP (23). Given the fact that Castleman disease has an extremely low incidence in the general population, cases of PNP with Castleman disease are highly frequent. A minor fraction of neoplasms associated with PNP represents non-hematologic neoplasms, including neoplasms originating from the thymus (e.g., thymoma), sarcoma, malignant melanoma, and various epithelial-origin carcinomas (e.g., adenocarcinoma and squamous cell carcinoma) (10, 14, 20, 24, 25). Some cases of PNP were diagnosed before an underlying malignancy was detected (26–28). Accordingly, PNP might be a marker for occult malignancy.

### Autoantibodies

**Figure 2:**
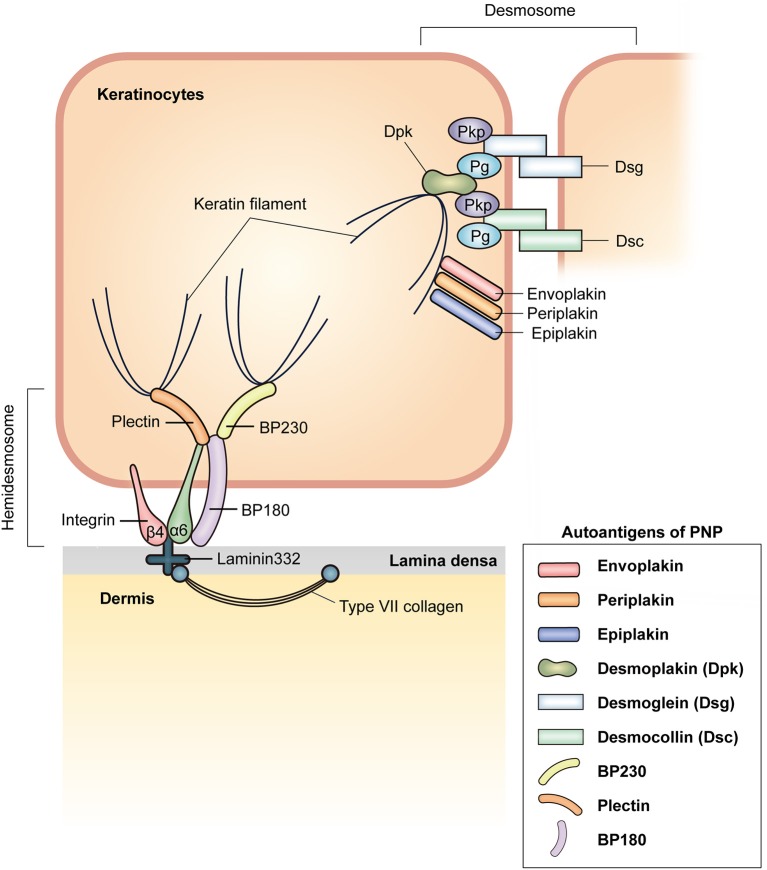
Schematic representation of a membrane-associated adhesive junction in the epidermis and autoantigens in PNP. Keratinocytes in the epidermis are connected via desmosomes. Desmosomal cadherins, desmoglein (Dsg) and desmocollin (Dsc), are transmembrane proteins that form hetero- or homodimers in the intercellular area. At the cytoplasmic side of the desmosome, plakophilin (Pkp) and plakoglobin (Pg) bind to intracellular domains of desmosomal cadherins. Desmoplakin (Dpk) interacts with Pkp, Pg, and keratin filaments. Envoplakin, periplakin, and epiplakin serve to link keratin filaments and the plasma membrane. Desmosomal components known to act as autoantigens in PNP are envoplakin, periplakin, epiplakin, Dpk, Dsg, and Dsc. Hemidesmosomes anchor the epidermis to the dermis. Plectin and BP230, which connect keratin filaments, bind to α6β4 integrin and BP180, which are transmembrane proteins in hemidesmosomes. α6β4 integrin binds to laminin 332, which interacts with type VII collagen in the dermis. Autoantibodies against BP230, BP180, and plectin can be observed in PNP.

PNP is characterized by the production of autoantibodies against various target antigens, mainly plakin family proteins (Figure 2). The plakin family is defined by the presence of a plakin and/or plakin repeat domain and function as linker proteins that link cytoskeletal networks to each other and to membrane-associated adhesive junctions, such as desmosomes and hemidesmosomes. The seven plakin family members include desmoplakins (Dpks: Dpk1 and Dpk2), plectin, BP230, microtubule-actin cross-linking factor 1, envoplakin, periplakin, and epiplakin (29). The most characteristic and consistently recognized plakin antigens in PNP are envoplakin (30) and periplakin (31). BP230, Dpks, epiplakin, and plectin are also frequently recognized as target antigens in PNP (31, 32). In addition, BP180 (33), p200 protein (34), desmosomal cadherins such as desmogleins (Dsgs: Dsg1 and Dsg3) (35) and desmocollins (Dscs: Dsc1, Dsc2, and Dsc3) (11), as well as the protease inhibitor alpha-2-macroglobulin-like antigen-1 (A2ML1) (36) are targeted in PNP (Figure 2).

## Diagnosis

### Histology

**Figure 3:**
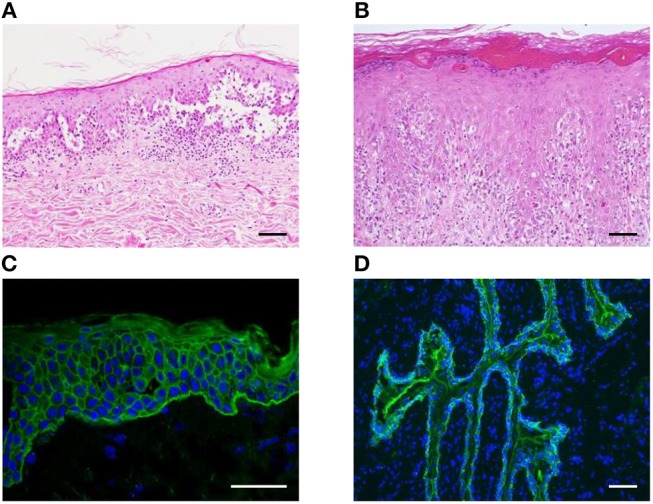
Histopathological and immunofluorescent findings of PNP. **(A,B)** Suprabasal acantholysis **(A)** and interface dermatitis with scattered dyskeratotic cells **(B)** are observed in PNP skin lesions (scale bar, 100 μm). **(C,D)** Using indirect immunofluorescence studies, IgG deposition on the intercellular spaces of keratinocytes and the dermo–epidermal junction **(C)** and on the surface of rat bladder epithelial cells **(D)** is found (scale bar, 100 μm).

As PNP has two major clinical phenotypes, i.e., blisters and lichenoid eruptions, pathologic findings are present as acantholytic blisters and interface dermatitis, depending on the clinical features (21). In blisters, suprabasal acantholytic separations with sparse inflammatory infiltrate are observed (Figure 3A), whereas lichenoid interface changes with a dense mononuclear immune cell infiltration in dermo–epidermal junction are observed in erythematous maculopapular lesions (Figure 3B). In addition, blisters and interface dermatitis sometimes coappear in the same lesion.

### Immunofluorescence

Immunofluorescence is a useful technique in the diagnosis of PNP. In direct immunofluorescence of the mucocutaneous lesions, IgG autoantibodies and/or complement deposition is observed in the epidermal intercellular spaces and/or along the basement membrane zone (4). Circulating autoantibodies can be found by indirect immunofluorescence (IIF) assays using human skin (Figure 3C), monkey or guinea pig esophagus, or other substrates, including rat bladder, myocardium, and lung. In particular, the bladder is rich in plakins such as envoplakin, periplakin, and Dpk but lacks Dsgs. Therefore, despite its relatively low sensitivity (86%), IIF using rat bladder is a highly specific (98%) method to differentiate PNP from other pemphigus that does not harbor anti-plakin autoantibodies (Figure 3D) (4, 37).

### Use of Antigen to Detect Autoantibodies

Immunoblotting is considered the gold standard for diagnosis of PNP (4), and immunoprecipitation and IIF using rat bladder are useful for diagnostic accuracy of PNP (4, 38). Immunoblot analysis using epidermal extracts has been used to detect 210 kDa envoplakin and 190 kDa periplakin, which are highly sensitive and specific for PNP (4). Immunoprecipitation can detect antibodies against multiple epidermal antigens, including plakin family proteins and the 170 kDa A2ML1 protein (36, 39).

Enzyme-linked immunosorbent assays (ELISAs) for envoplakin and periplakin have been developed for PNP diagnosis (38, 40–42). A series of studies using epitope mapping showed that ELISAs using the recombinant N-terminal domain and the linker subdomain of envoplakin and the linker subdomain of periplakin exhibit 75% sensitivity and 92–99% specificity (38, 40–42). ELISA is a useful technique for detecting circulating autoantibodies in PNP, especially those against Dsgs and Dscs. Approximately 80% of patients with PNP have circulating anti-Dsg3 IgG, and other autoantibodies against desmosomal cadherins (e.g., Dsg1, Dsc1, Dsc2, and Dsc3) have been detected in some patients with PNP (19–42%) (11). Moreover, autoantibodies against BP180 are detected in ~40% of PNP sera (33).

## Management and Prognosis

The treatment of PNP is challenging; however, PNP cases associated with benign tumors, such as localized Castleman disease and benign thymoma, generally improve or achieve complete remission within 1–2 years after complete tumor resection (43). However, in PNP with malignant neoplasms, reducing the tumor burden does not lead to control of the disease, and a consensus regarding the best therapeutic regimen for treatment has yet to be established. The most widely used treatment for PNP is systemic corticosteroids, but many patients with PNP do not show a good response with corticosteroids alone (44). Systemic corticosteroids are also used with other immunosuppressive agents, including cyclosporine, cyclophosphamide, azathioprine, and mycophenolate mofetil (45). However, the clinical efficacy of combination therapy varies depending on the underlying neoplasm. Cutaneous lesions usually improve after treatment with these immunosuppressive drugs, whereas mucositis is often refractory to these treatments (45).

Intravenous immunoglobulin and plasmapheresis are commonly used for the treatment of autoimmune bullous diseases. Both treatments have shown promising effects in the treatment of PNP (46, 47). B cell-targeting agents have also been used in PNP. Rituximab, a monoclonal anti-CD20 antibody, depletes mature CD20^+^ B cells, and ibrutinib, a Bruton's tyrosine kinase inhibitor, inhibits B cell signaling. Rituximab and ibrutinib produce different outcomes among PNP patients, but generally, the responses are good (48–50). In contrast to humoral immunity, cellular immunity cannot be controlled by these treatment options, which may explain why complete remission is not achieved in all PNP patients with these treatments. Therefore, therapeutic strategies for controlling both humoral and cellular autoimmunity should be considered in order to achieve complete remission in PNP. Alemtuzumab is a monoclonal antibody against CD52, which is expressed on most T and B lymphocytes. Alemtuzumab was shown to be effective in PNP patients refractory to various treatments, including corticosteroids, but it has only been administrated in a few cases of PNP with hematologic malignancies (51, 52). Tocilizumab, a monoclonal antibody against IL-6R, was found to rapidly improved mucositis, but not bronchiolitis obliterans, in two cases of PNP (53).

Prognosis of PNP is poor, and mortality is high, with a 5-year overall survival rate of only 38%, although prognosis largely depends on the nature of the underlying malignancy (2, 44). The course of PNP is not correlated with that of the associated malignancy (2). Mortality usually results from severe infection due to the immunosuppressive therapy, associated malignancy, and bronchiolitis obliterans (2, 11, 21, 44). Bronchiolitis obliterans may cause respiratory failure, leading to a fatal outcome. Indeed, one study showed that bronchiolitis obliterans and toxic epidermal necrolysis-like clinical feature are independent risk factors for death in PNP (54). Similar to mucositis, bronchiolitis obliterans is resistant to therapy, and lung transplantation is the last therapeutic option for respiratory failure (55).

## Immunopathology of PNP

### Humoral Immunity

**Figure 4:**
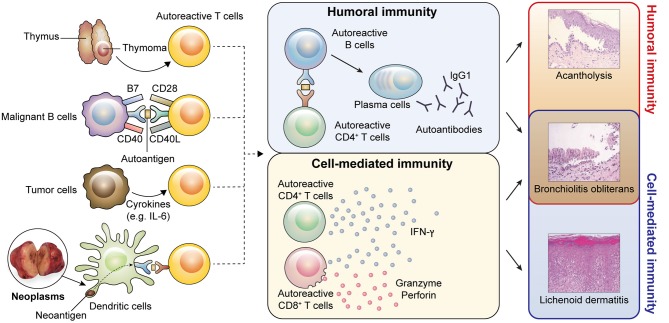
Pathophysiology of PNP. Possible models of autoreactive T cell generation caused by neoplasms are shown. (1) Neoplasms originating from the thymus may interfere with the negative selection process during central tolerance, resulting in survival of autoreactive T cells. (2) Tumor cells originating from B cells can act as antigen-presenting cells. Tumor cells may present self-antigens and provide co-activating signals to autoreactive naïve T cells. Thus, autoreactive T cells can escape anergy. (3) Tumor cells secrete cytokines, such as IL-6, which can drive the conversion of regulatory T cells (Tregs) into effector T cells. A lack of Tregs may promote the activation of autoreactive T cells. (4) Neoantigens derived from neoplasms may act as antigens to autoreactive T cells. Activated autoreactive T cells induce both humoral and cell-mediated immunity. In humoral immunity, autoreactive B cells interact with autoreactive T cells through cognate antigens and differentiate into plasma cells, which produce IgG1 autoantibodies. Humoral autoimmunity contributes to bronchiolitis obliterans and acantholysis presenting as blisters. In cell-mediated immunity, autoreactive CD4^+^ and CD8^+^ T cells produce interferon-γ (IFN-γ) and autoreactive CD8^+^ T cells secrete cytotoxic molecules, such as granzyme and perforin. These immune reactions induce bronchiolitis obliterans and lichenoid dermatitis. (A histologic image of bronchiolitis obliterans were adopted from Nousari et al. (18). The permission was obtained from the authors for reproduction).

As desmosomal cadherins are the only desmosomal components exposed on the cell surface, it was first suspected that autoantibodies against desmosomal cadherins cause the suprabasal acantholytic blisters in PNP (Figure 4). This was clearly supported by a study using neonatal mice injected with IgGs from PNP sera (35). In this study, mice given IgGs depleted with anti-Dsg IgGs were protected from blisters, whereas anti-Dsg3 IgGs caused acantholytic blisters (35, 56). However, some patients with PNP having suprabasal acantholytic mucosal and skin blisters do not have circulating anti-Dsg autoantibodies (21, 57). This phenomenon is also observed in pemphigus, one of the autoimmune bullous mucocutaneous diseases characterized by anti-Dsg autoantibodies. In some cases showing the pemphigus phenotype, blisters can develop because of autoantibodies against Dsc3 but not against Dsgs (58). These findings confirm that the mechanism of acantholysis in PNP varies among patients. A recent study showed that antibodies to A2ML1, which act as a protease inhibitor, decrease the adhesion of cultured normal human keratinocytes by activating plasmin. This suggests that anti-A2ML1 autoantibodies from PNP sera may contribute to the induction of acantholysis (36). Furthermore, it remains to be determined whether anti-plakin family antibodies play a role in the induction of acantholytic blisters in PNP (59). Thus, further studies are needed to clarify the exact role of autoantibodies in the development of acantholytic blisters in PNP.

Bronchiolitis obliterans was first examined in studies using bronchus biopsy specimens from PNP patients (18, 60). In the bronchial epithelium, ciliated basal cells adhered to the lamina propria, whereas ciliated columnar cells are separated (18, 60). In line with the histological findings, linear deposition of IgG was observed in the intercellular spaces of respiratory epithelial cells as well as the basement membrane zone (18). These findings provided evidence that humoral immunity can contribute to the development of bronchiolitis obliterans in PNP (Figure 4). However, it is still uncertain which types of autoantibodies are pathogenic in bronchiolitis obliterans. Importantly, desmosomal cadherins are differentially expressed between the skin and bronchus. In particular, Dsg1 and Dsg3, expressed in the skin and mucosal epidermis, are not expressed in normal respiratory epithelium (18). However, Dsg3 can be ectopically expressed in the lung in the case of squamous metaplasia in response to inflammation (61). Thus, anti-Dsg3 antibody might contribute to the pathogenesis of bronchiolitis obliterans. In a recent study, mice treated with anti-epiplakin antibodies showed loss of cell–cell adhesion in the respiratory epithelium (32), suggesting that anti-epiplakin antibody may play a pathogenic role in bronchiolitis obliterans, although epiplakin is located within the subcellular area of epithelial cells (62).

**Figure 5:**
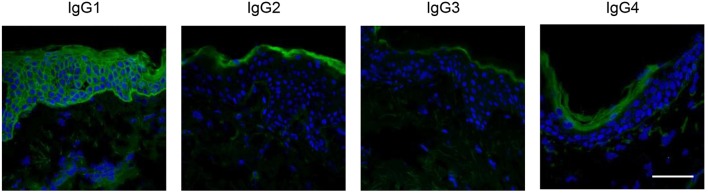
IgG isotypes of autoantibodies in PNP. Indirect immunofluorescence of serum from a patient with PNP was performed using fluorescence-labeled anti-IgG1, IgG2, IgG3, and IgG4 antibodies. The IgG1 isotype autoantibodies were predominantly detected (scale bar, 100 μm).

Human IgG is divided into four subclasses: IgG1, IgG2, IgG3, and IgG4. Among the IgG subclasses, anti-Dsg IgG1 is dominant in the sera of patients with PNP (63, 64), whereas anti-Dsg IgG4 is pathogenic in patients with pemphigus vulgaris and pemphigus foliaceus (65) (Figure 5). In human immunity, IgG1 is the main isotype in Th1 immunity, whereas IgG4 is mainly secreted during Th2 response. Therefore, the above results suggest that the Th1 response might be dominant in PNP. In addition, anti-Dsg3 antibody from PNP sera reacts with all five extracellular (EC) subdomains of human Dsg3, whereas anti-Dsg3 antibody from pemphigus vulgaris sera mainly binds to EC1 and EC2 domains (63). Pathogenic epitopes of Dsg3 are also different between PNP and pemphigus vulgaris. Pathogenic monoclonal antibodies from PNP bind to EC2 and EC3 domains (56), in contrast to those of pemphigus vulgaris binding to EC1 domain (66). The differences in Dsg epitopes and subclass distribution reflect the difference in the mechanisms mediating autoimmunity between PNP and pemphigus.

### Cellular Immunity

The presence of lichenoid dermatitis in PNP indicates that cell-mediated immune mechanisms play a critical role in its development (67, 68) (Figure 4). CD8^+^ T cell infiltration and apoptotic keratinocytes are frequently observed in the epidermis of PNP (6, 69), suggesting that autoreactive CD8^+^ T cells targeting epidermal components contribute to the formation of lichenoid dermatitis. CD56^+^ cells are also detected in lichenoid dermatitis (6), but further studies are needed to characterize these cells since CD56 is expressed on CD8^+^ T cells as well as natural killer cells. With regard to CD4^+^ T cell-mediated immunity, adoptive transfer of Dsg3-specific CD4^+^ T cells into *RAG2*^−/−^ mice was found to cause interface dermatitis as a result of cell-mediated immunity, and interferon-γ from CD4^+^ T cells was shown as a crucial inducer of this interface dermatitis (70). Lichenoid dermatitis may be the only sign of PNP or may develop before blisters appear (68, 71, 72). Thus, this suggests that lichenoid inflammation induced by cell-mediated immunity might lead to exposure of self-antigens, such as plakins, to the immune system, thereby inducing autoantibody production.

In addition to mucocutaneous lesions, marked infiltration of CD8^+^ T cells is observed in PNP-associated bronchiolitis obliterans and in the lungs of *DSG3*^−/−^ mice injected with IgGs from PNP sera (6, 73). These findings implicate CD8^+^ T cell-mediated immunity in the pathogenesis of bronchiolitis obliterans (Figure 4). Moreover, adoptive transfer of Dsg3-specific CD4^+^ T cells in *RAG2*^−/−^ mice induced pulmonary inflammation and ectopic Dsg3 expression (61) (Figure 4). Therefore, both humoral and cell-mediated immunity may be involved in the development of bronchiolitis obliterans in PNP, although further studies will be required to understand the exact pathophysiological mechanisms underlying bronchiolitis obliterans.

## Potential Pathomechanisms of Paraneoplastic Autoimmunity

### Breakdown of Central Tolerance

T cells develop in the thymus and undergo positive and negative selection during development before entering the periphery. During positive selection in the thymic cortex, T cells that cannot interact with self-peptide-bound major histocompatibility complex (MHC) molecules are removed. Autoreactive T cells bearing TCR with high affinity to self-peptide-bound MHC molecules are removed during negative selection in the thymic medulla. In this process, tissue-specific antigens are expressed in the medullary thymic epithelial cells through the action of factors such as autoimmune regulator (Aire) (74). If the negative selection process cannot be precisely controlled owing to the presence of a tumor in the thymus, autoreactive T cells may escape central tolerance and promote autoimmunity in the peripheral area.

Thymoma is a neoplasm commonly associated with PNP. PNP patients with benign thymoma are usually cured after complete tumor resection (21, 75). Thymoma is well-known to induce an autoimmune response (76). Indeed, other autoimmune diseases, including myasthenia gravis, can occur in patients with thymoma (76), and PNP associated with thymoma is often accompanied by myasthenia gravis (21, 77). Thymoma has no or reduced medullary portions and is defective in the expression of Aire (78, 79). T cells from *AIRE*^−/−^ mice induced the production of anti-Dsg3 IgG antibodies when interacting with *DSG3*^−/−^ B cells (80), and Aire-dependent medullary thymic epithelial cells expressed Dsgs (81). However, autoimmune polyendocrinopathy-candidiasis-ectodermal dystrophy, a human hereditary disease with Aire deficiency, neither presents anti-Dsg and anti-BPAG1 antibodies nor the clinical features of PNP (82–84). Recently, in a patient with thymoma expressing Aire, the condition manifested as pemphigus foliaceus with anti-Dsg1 autoantibody (85). These results suggest that Aire may not be the only factor regulating central tolerance in PNP (Figure 4). Given that thymic factors other than Aire (e.g., Fezf2) also contribute to the negative selection (86), the mechanism of breakdown of central tolerance in PNP must be further clarified.

### Breakdown of Peripheral Tolerance

Even if thymic selection yields high-purity T cells recognizing foreign antigens, some self-reactive T cells escape to the periphery. However, peripheral tolerance prevents the activation of self-reactive T cells in peripheral tissues via several mechanisms, including T cell anergy and deletion and suppression by regulatory T cells (Tregs). T cell anergy, a long-lived hyporesponsive state of T cells, occurs when T cells engage MHC molecules on antigen presenting cells (APCs) in the absence of costimulatory signals (87). T cell deletion entails T cell apoptosis due to repeated stimulation of T cells without costimulation (88).

CD28, one of the classic costimulatory molecules in T cells, interacts with its ligands (CD80 [B7-1] and CD86 [B7-2]) expressed on professional APCs. In contrast to solid tumors, lymphomas derived from B cells express CD80 or/and CD86 (89–92), which induce T cell proliferation and prevent T cell anergy (90). CLL B cells lack CD80 and CD86 but upregulate CD80 and CD86 after stimulation, thereby presenting antigens and activating T cells (93, 94). Moreover, lymph node-derived CLL cells show higher CD80 and CD86 expression than circulating CLL cells (95). These results suggest that tumor cells derived from B cells have functional costimulatory molecules. Thus, self-reactive T cells might be activated after escape from peripheral tolerance by mechanisms such as anergy and deletion (Figure 4).

Tregs have a critical role in regulating T cell activation in peripheral tolerance. Cytotoxic T lymphocyte-associated antigen-4 (CTLA-4), a structural homolog of CD28, is expressed on Tregs and has a substantially higher affinity for CD80 and CD86 than does CD28. CTLA-4 competitively inhibits CD28-CD80/CD86 signaling and downregulates CD80 and CD86 expression, so that Tregs induce self-reactive T cell anergy and inactivation (96). Ipilimumab, a CTLA-4-blocking antibody, aggravates pre-existing autoimmune diseases (97). Tregs are heterogenous and can be unstable, depending on the environment (98). A thymically derived Treg cell population generally maintains its suppressive activity, whereas a peripherally derived Treg cell population can change its functional properties under inflammatory conditions (99). Although the role of Tregs in PNP has not been studied, recent studies in *FOXP3*^−/−^ scurfy mice revealed that the absence of Tregs leads to autoimmune bullous skin diseases mediated by anti-BP230 antibodies (100, 101). Similar to the findings of the mouse study, bullous pemphigoid, characterized by anti-BP180 and anti-BP230 autoantibodies, reportedly developed in a pediatric patient with immune dysregulation, polyendocrinopathy, enteropathy, and X-linked (IPEX) syndrome caused by *FOXP3* mutation (102). Thus, a Treg imbalance might lead to the induction of paraneoplastic autoimmunity.

The pro-inflammatory cytokine interleukin (IL)-6 is the major extrinsic factor inhibiting Treg differentiation (103, 104). *IL6*^−/−^ mice or mice treated with IL-6R blocking antibody exhibit increased frequencies of Tregs and are resistant to various autoimmune diseases (105, 106). Besides Treg differentiation, IL-6 inhibits FoxP3 expression and the suppressive function of Tregs (107). Further, IL-6 promotes the differentiation and function of T follicular helper cells, which interact with B cells and help B cell proliferation, differentiation, and isotype switching (108). A majority of PNP cases showed markedly elevated serum IL-6 levels (109, 110), and recent studies showed that IL-6 is a major driver of disease progression in idiopathic multicentric Castleman disease, which has a substantially higher incidence in PNP than that in other neoplasms (111). Taken together, these results imply that IL-6 might be a crucial inducer of paraneoplastic autoimmunity, although additional studies are required to substantiate the relationship between IL-6 and autoimmunity in PNP (Figure 4).

### Molecular Mimicry

PNP might also be caused by an antitumor immune response. Tumor-specific neoantigens result from the mutation of tumors. T cells in response to neoantigens can cross-react with self-antigens derived from normal epithelial proteins and thereby induce autoimmunity due to molecular mimicry. Neoantigens mimicking self-antigens derived from desmosomal and hemidesmosomal proteins have not been investigated in neoplasms to date, although studies have shown that several proteins including Dsg3, BP180, BP230, and α6β4 integrin are overexpressed in epithelial-origin carcinoma (112–115). Once an autoimmune response against a self-antigen starts, tissue damage may propagate the activation of adaptive immune cells specific for other self-antigens, which is called epitope spreading (116). The concept of epitope spreading may explain why autoantibodies targeting multiple self-antigens are detected in individuals with PNP.

## Future Directions

Because it is such a rare disease, PNP has been poorly understood to date. Although our understanding of PNP is gradually increasing, the pathogenesis and etiology of this disease remain unknown. Moreover, there is a lack of effective treatment options for PNP. Additional human and animal studies will be necessary to investigate the role of anti-plakin autoantibodies in disease manifestation and the mechanism of bronchiolitis obliterans. The causes of PNP might be heterogeneous, depending on the associated malignancies; therefore, various basic approaches are needed to comprehend the breakdown of immune tolerance in PNP. Presently, there is no consensus of diagnostic criteria for this disease. Thus, large-scale clinical studies are needed to optimize the diagnostic algorithm and to develop additional effective treatment strategies to suppress the autoimmune response.

## Author Contributions

JK wrote and edited the manuscript. S-CK edited the manuscript.

### Conflict of Interest Statement

The authors declare that the research was conducted in the absence of any commercial or financial relationships that could be construed as a potential conflict of interest.

## References

[1] Anhalt GJ Kim SC Stanley JR Korman NJ Jabs DA Kory M. Paraneoplastic pemphigus. An autoimmune mucocutaneous disease associated with neoplasia. N Engl J Med. (1990) 323:1729–35. 10.1056/NEJM199012203232503 2247105

[2] Anhalt GJ. Paraneoplastic pemphigus. J Investig Dermatol Symp Proc. (2004) 9:29–33. 10.1111/j.1087-0024.2004.00832.x 14870982

[3] Zhu X Zhang B. Paraneoplastic pemphigus. J Dermatol. (2007) 34:503–11. 10.1111/j.1346-8138.2007.00322.x 17683379

[4] Joly P Richard C Gilbert D Courville P Chosidow O Roujeau JC. Sensitivity and specificity of clinical, histologic, and immunologic features in the diagnosis of paraneoplastic pemphigus. J Am Acad Dermatol. (2000) 43:619–26. 10.1067/mjd.2000.107488 11004616

[5] Zimmermann J Bahmer F Rose C Zillikens D Schmidt E. Clinical and immunopathological spectrum of paraneoplastic pemphigus. J Dtsch Dermatol Ges. (2010) 8:598–606. 10.1111/j.1610-0387.2010.07380.x 20180886

[6] Nguyen VT Ndoye A Bassler KD Shultz LD Shields MC Ruben BS. Classification, clinical manifestations, and immunopathological mechanisms of the epithelial variant of paraneoplastic autoimmune multiorgan syndrome: a reappraisal of paraneoplastic pemphigus. Arch Dermatol. (2001) 137:193–206. 11176692

[7] Paolino G Didona D Magliulo G Iannella G Didona B Mercuri SR. Paraneoplastic pemphigus: insight into the autoimmune pathogenesis, clinical features and therapy. Int J Mol Sci. (2017) 18:2532. 10.3390/ijms18122532 29186863 PMC5751135

[8] Liu Q Bu DF Li D Zhu XJ. Genotyping of HLA-I and HLA-II alleles in Chinese patients with paraneoplastic pemphigus. Br J Dermatol. (2008) 158:587–91. 10.1111/j.1365-2133.2007.08361.x 18070207

[9] Martel P Loiseau P Joly P Busson M Lepage V Mouquet H. Paraneoplastic pemphigus is associated with the DRB1^*^03 allele. J Autoimmun. (2003) 20:91–5. 10.1016/S0896-8411(02)00092-6 12604316

[10] Kaplan I Hodak E Ackerman L Mimouni D Anhalt GJ Calderon S. Neoplasms associated with paraneoplastic pemphigus: a review with emphasis on non-hematologic malignancy and oral mucosal manifestations. Oral Oncol. (2004) 40:553–62. 10.1016/j.oraloncology.2003.09.020 15063382

[11] Ohzono A Sogame R Li X Teye K Tsuchisaka A Numata S. Clinical and immunological findings in 104 cases of paraneoplastic pemphigus. Br J Dermatol. (2015) 173:1447–52. 10.1111/bjd.14162 26358412

[12] Lim JM Lee SE Seo J Kim DY Hashimoto T Kim SC. Paraneoplastic pemphigus associated with a malignant thymoma: a case of persistent and refractory oral ulcerations following thymectomy. Ann Dermatol. (2017) 29:219–22. 10.5021/ad.2017.29.2.219 28392652 PMC5383750

[13] Lam S Stone MS Goeken JA Massicotte SJ Smith AC Folberg R. Paraneoplastic pemphigus, cicatricial conjunctivitis, and acanthosis nigricans with pachydermatoglyphy in a patient with bronchogenic squamous cell carcinoma. Ophthalmology. (1992) 99:108–13. 10.1016/S0161-6420(92)32030-5 1741121

[14] Lee SE Kim SC Paraneoplastic pemphigus. Dermatol Sinica. (2010) 28:1–14. 10.1016/S1027-8117(10)60001-8

[15] Lee SE Kim HR Hashimoto T Kim SC. Paraneoplastic pemphigus developed shortly after resection of follicular dendritic cell sarcoma. Acta Derm Venereol. (2008) 88:410–2. 10.2340/00015555-0446 18709322

[16] Kim SC Chang SN Lee IJ Park SD Jeong ET Lee CW. Localized mucosal involvement and severe pulmonary involvement in a young patient with paraneoplastic pemphigus associated with Castleman's tumour. Br J Dermatol. (1998) 138:667–71. 10.1046/j.1365-2133.1998.02183.x 9640377

[17] Bialy-Golan A Brenner S Anhalt GJ. Paraneoplastic pemphigus: oral involvement as the sole manifestation. Acta Derm Venereol. (1996) 76:253–4. 8800320 10.2340/0001555576253254

[18] Nousari HC Deterding R Wojtczack H Aho S Uitto J Hashimoto T. The mechanism of respiratory failure in paraneoplastic pemphigus. N Engl J Med. (1999) 340:1406–10. 10.1056/NEJM199905063401805 10228191

[19] Lee J Bloom R Amber KT. A systematic review of patients with mucocutaneous and respiratory complications in paraneoplastic autoimmune multiorgan syndrome: castleman's disease is the predominant malignancy. Lung. (2015) 193:593–6. 10.1007/s00408-015-9732-8 25903794

[20] Lehman VT Barrick BJ Pittelkow MR Peller PJ Camilleri MJ Lehman JS. Diagnostic imaging in paraneoplastic autoimmune multiorgan syndrome: retrospective single site study and literature review of 225 patients. Int J Dermatol. (2015) 54:424–37. 10.1111/ijd.12603 25069905

[21] Choi Y Nam KH Lee JB Lee JY Ihm CW Lee SE. Retrospective analysis of 12 Korean patients with paraneoplastic pemphigus. J Dermatol. (2012) 39:973–81. 10.1111/j.1346-8138.2012.01655.x 22938021

[22] Wang J Zhu X Li R Tu P Wang R Zhang L. Paraneoplastic pemphigus associated with Castleman tumor: a commonly reported subtype of paraneoplastic pemphigus in China. Arch Dermatol. (2005) 141:1285–93. 10.1001/archderm.141.10.1285 16230567

[23] Mimouni D Anhalt GJ Lazarova Z Aho S Kazerounian S Kouba DJ. Paraneoplastic pemphigus in children and adolescents. Br J Dermatol. (2002) 147:725–32. 10.1046/j.1365-2133.2002.04992.x 12366419

[24] Amber KT Valdebran M Grando SA. Paraneoplastic autoimmune multiorgan syndrome (PAMS): beyond the single phenotype of paraneoplastic pemphigus. Autoimmun Rev. (2018) 17:1002–10. 10.1016/j.autrev.2018.04.008 30103046

[25] Hong WJ Lee SE Chang SE Hashimoto T Kim SC. Paraneoplastic pemphigus associated with metastatic lymphoepithelioma-like carcinoma originating from the thyroid gland. Br J Dermatol. (2015) 172:831–4. 10.1111/bjd.13334 25112893

[26] Ostezan LB Fabre VC Caughman SW Swerlick RA Korman NJ Callen JP. Paraneoplastic pemphigus in the absence of a known neoplasm. J Am Acad Dermatol. (1995) 33(2 Pt 1):312–5. 10.1016/0190-9622(95)90269-4 7622665

[27] Verrini A Cannata G Cozzani E Terracini M Parodi A Rebora A. A patient with immunological features of paraneoplastic pemphigus in the absence of a detectable malignancy. Acta Derm Venereol. (2002) 82:382–4. 10.1080/000155502320624177 12430743

[28] Park GT Lee JH Yun SJ Lee SC Lee JB. Paraneoplastic pemphigus without an underlying neoplasm. Br J Dermatol. (2007) 156:563–6. 10.1111/j.1365-2133.2006.07605.x 17300250

[29] Bouameur JE Favre B Borradori L. Plakins, a versatile family of cytolinkers: roles in skin integrity and in human diseases. J Invest Dermatol. (2014) 134:885–94. 10.1038/jid.2013.498 24352042

[30] Kim SC Kwon YD Lee IJ Chang SN Lee TG. cDNA cloning of the 210-kDa paraneoplastic pemphigus antigen reveals that envoplakin is a component of the antigen complex. J Invest Dermatol. (1997) 109:365–9. 10.1111/1523-1747.ep12336235 9284106

[31] Mahoney MG Aho S Uitto J Stanley JR. The members of the plakin family of proteins recognized by paraneoplastic pemphigus antibodies include periplakin. J Invest Dermatol. (1998) 111:308–13. 10.1046/j.1523-1747.1998.00279.x 9699735

[32] Tsuchisaka A Numata S Teye K Natsuaki Y Kawakami T Takeda Y. Epiplakin is a paraneoplastic pemphigus autoantigen and related to bronchiolitis obliterans in japanese patients. J Invest Dermatol. (2016) 136:399–408. 10.1038/JID.2015.408 26802236

[33] Tsuchisaka A Kawano H Yasukochi A Teye K Ishii N Koga H. Immunological and statistical studies of anti-BP180 antibodies in paraneoplastic pemphigus. J Invest Dermatol. (2014) 134:2283–7. 10.1038/jid.2014.151 24658507

[34] Oh SJ Lee SE Hashimoto T Kim SC. A case of paraneoplastic pemphigus associated with Castleman disease reacting with multiple autoantigens, including the p200 protein. Br J Dermatol. (2016) 174:930–2. 10.1111/bjd.14293 27115591

[35] Amagai M Nishikawa T Nousari HC Anhalt GJ Hashimoto T. Antibodies against desmoglein 3 (pemphigus vulgaris antigen) are present in sera from patients with paraneoplastic pemphigus and cause acantholysis *in vivo* in neonatal mice. J Clin Invest. (1998) 102:775–82. 10.1172/JCI3647 9710446 PMC508940

[36] Numata S Teye K Tsuruta D Sogame R Ishii N Koga H. Anti-alpha-2-macroglobulin-like-1 autoantibodies are detected frequently and may be pathogenic in paraneoplastic pemphigus. J Invest Dermatol. (2013) 133:1785–93. 10.1038/jid.2013.65 23407400

[37] Liu AY Valenzuela R Helm TN Camisa C Melton AL Bergfeld WF. Indirect immunofluorescence on rat bladder transitional epithelium: a test with high specificity for paraneoplastic pemphigus. J Am Acad Dermatol. (1993) 28(5 Pt 1):696–9. 10.1016/0190-9622(93)70095-B 7684408

[38] Poot AM Diercks GF Kramer D Schepens I Klunder G Hashimoto T. Laboratory diagnosis of paraneoplastic pemphigus. Br J Dermatol. (2013) 169:1016–24. 10.1111/bjd.12479 23796242

[39] Hashimoto T Amagai M Watanabe K Chorzelski TP Bhogal BS Black MM. Characterization of paraneoplastic pemphigus autoantigens by immunoblot analysis. J Invest Dermatol. (1995) 104:829–34. 10.1111/1523-1747.ep12607012 7738363

[40] Nagata Y Karashima T Watt FM Salmhofer W Kanzaki T Hashimoto T. Paraneoplastic pemphigus sera react strongly with multiple epitopes on the various regions of envoplakin and periplakin, except for the c-terminal homologous domain of periplakin. J Invest Dermatol. (2001) 116:556–63. 10.1046/j.1523-1747.2001.01263.x 11286623

[41] Probst C Schlumberger W Stocker W Recke A Schmidt E Hashimoto T. Development of ELISA for the specific determination of autoantibodies against envoplakin and periplakin in paraneoplastic pemphigus. Clin Chim Acta. (2009) 410:13–8. 10.1016/j.cca.2009.08.022 19737550

[42] Wang X Chen T Zhao J Peng Y Chen X Tu P. Extremities of the N-terminus of envoplakin and C-terminus of its linker subdomain are major epitopes of paraneoplastic pemphigus. J Dermatol Sci. (2016) 84:24–9. 10.1016/j.jdermsci.2016.06.011 27427435

[43] Zhang J Qiao QL Chen XX Liu P Qiu JX Zhao H. Improved outcomes after complete resection of underlying tumors for patients with paraneoplastic pemphigus: a single-center experience of 22 cases. J Cancer Res Clin Oncol. (2011) 137:229–34. 10.1007/s00432-010-0874-z 20390428 PMC11827967

[44] Leger S Picard D Ingen-Housz-Oro S Arnault JP Aubin F Carsuzaa F. Prognostic factors of paraneoplastic pemphigus. Arch Dermatol. (2012) 148:1165–72. 10.1001/archdermatol.2012.1830 22801794

[45] Frew JW Murrell DF. Current management strategies in paraneoplastic pemphigus (paraneoplastic autoimmune multiorgan syndrome). Dermatol Clin. (2011) 29:607–12. 10.1016/j.det.2011.06.016 21925005

[46] Nanda M Nanda A Al-Sabah H Dvorak R Alsaleh QA. Paraneoplastic pemphigus in association with B-cell lymphocytic leukemia and hepatitis C: favorable response to intravenous immunoglobulins and prednisolone. Int J Dermatol. (2007) 46:767–9. 10.1111/j.1365-4632.2007.03225.x 17614814

[47] Izaki S Yoshizawa Y Kitamura K Kato H Hashimoto H Korman NJ. Paraneoplastic pemphigus: potential therapeutic effect of plasmapheresis. Br J Dermatol. (1996) 134:987–9. 10.1111/j.1365-2133.1996.tb06349.x 8736359

[48] Vezzoli P Berti E Marzano AV. Rationale and efficacy for the use of rituximab in paraneoplastic pemphigus. Expert Rev Clin Immunol. (2008) 4:351–63. 10.1586/1744666X.4.3.351 20476925

[49] Lee A Sandhu S Imlay-Gillespie L Mulligan S Shumack S. Successful use of Bruton's kinase inhibitor, ibrutinib, to control paraneoplastic pemphigus in a patient with paraneoplastic autoimmune multiorgan syndrome and chronic lymphocytic leukaemia. Australas J Dermatol. (2017) 58:e240–e242. 10.1111/ajd.12615 28295171

[50] Ito Y Makita S Maeshima AM Hatta S Suzuki T Yuda S. Paraneoplastic pemphigus associated with B-cell chronic lymphocytic leukemia treated with ibrutinib and rituximab. Intern Med. (2018) 57:2395–8. 10.2169/internalmedicine.0578-17 29526963 PMC6148183

[51] Hohwy T Bang K Steiniche T Peterslund NA d'Amore F. Alemtuzumab-induced remission of both severe paraneoplastic pemphigus and leukaemic bone marrow infiltration in a case of treatment-resistant B-cell chronic lymphocytic leukaemia. Eur J Haematol. (2004) 73:206–9. 10.1111/j.1600-0609.2004.00280.x 15287918

[52] Bech R Baumgartner-Nielsen J Peterslund NA Steiniche T Deleuran M d'Amore F. Alemtuzumab is effective against severe chronic lymphocytic leukaemia-associated paraneoplastic pemphigus. Br J Dermatol. (2013) 169:469–72. 10.1111/bjd.12324 23517368

[53] Gu L Ye S. Tocilizumab cannot prevent the development of bronchiolitis obliterans in patients with castleman disease-associated paraneoplastic pemphigus. J Clin Rheumatol. (2018). [Epub ahead of print]. 10.1097/00124743-900000000-99360 29389687

[54] Ouedraogo E Gottlieb J de Masson A Lepelletier C Jachiet M Salle de Chou C. Risk factors for death and survival in paraneoplastic pemphigus associated with hematologic malignancies in adults. J Am Acad Dermatol. (2019) 80:1544–9. 10.1016/j.jaad.2018.03.043 30981429

[55] Chin AC Stich D White FV Radhakrishnan J Holterman MJ. Paraneoplastic pemphigus and bronchiolitis obliterans associated with a mediastinal mass: a rare case of Castleman's disease with respiratory failure requiring lung transplantation. J Pediatr Surg. (2001) 36:E22. 10.1053/jpsu.2001.28877 11733934

[56] Saleh MA Ishii K Yamagami J Shirakata Y Hashimoto K Amagai M. Pathogenic anti-desmoglein 3 mAbs cloned from a paraneoplastic pemphigus patient by phage display. J Invest Dermatol. (2012) 132:1141–8. 10.1038/jid.2011.449 22277944

[57] Inaoki M Kodera M Fujimoto A Nousari HC Anhalt GJ Takehara K Paraneoplastic pemphigus without antidesmoglein 3 or antidesmoglein 1 autoantibodies. Br J Dermatol. (2001) 144:610–3. 10.1046/j.1365-2133.2001.04095.x 11260026

[58] Mao X Nagler AR Farber SA Choi EJ Jackson LH Leiferman KM. Autoimmunity to desmocollin 3 in pemphigus vulgaris. Am J Pathol. (2010) 177:2724–30. 10.2353/ajpath.2010.100483 20952584 PMC2993297

[59] Li J Bu DF Huang YC Zhu XJ. Role of autoantibodies against the linker subdomains of envoplakin and periplakin in the pathogenesis of paraneoplastic pemphigus. Chin Med J. (2009) 122:486–95. 10.3760/cma.j.issn.0366-6999.2009.05.002 19323896

[60] Fullerton SH Woodley DT Smoller BR Anhalt GJ. Paraneoplastic pemphigus with autoantibody deposition in bronchial epithelium after autologous bone marrow transplantation. JAMA. (1992) 267:1500–2. 10.1001/jama.1992.03480110076037 1538540

[61] Hata T Nishimoto S Nagao K Takahashi H Yoshida K Ohyama M. Ectopic expression of epidermal antigens renders the lung a target organ in paraneoplastic pemphigus. J Immunol. (2013) 191:83–90. 10.4049/jimmunol.1203536 23729442

[62] Jang SI Kalinin A Takahashi K Marekov LN Steinert PM. Characterization of human epiplakin: RNAi-mediated epiplakin depletion leads to the disruption of keratin and vimentin IF networks. J Cell Sci. (2005) 118(Pt 4):781–93. 10.1242/jcs.01647 15671067

[63] Futei Y Amagai M Hashimoto T Nishikawa T. Conformational epitope mapping and IgG subclass distribution of desmoglein 3 in paraneoplastic pemphigus. J Am Acad Dermatol. (2003) 49:1023–8. 10.1016/S0190-9622(03)02160-1 14639380

[64] Brandt O Rafei D Podstawa E Niedermeier A Jonkman MF Terra JB. Differential IgG recognition of desmoglein 3 by paraneoplastic pemphigus and pemphigus vulgaris sera. J Invest Dermatol. (2012) 132:1738–41. 10.1038/jid.2012.1 22318391

[65] Futei Y Amagai M Ishii K Kuroda-Kinoshita K Ohya K Nishikawa T. Predominant IgG4 subclass in autoantibodies of pemphigus vulgaris and foliaceus. J Dermatol Sci. (2001) 26:55–61. 10.1016/S0923-1811(00)00158-4 11323221

[66] Payne AS Ishii K Kacir S Lin C Li H Hanakawa Y. Genetic and functional characterization of human pemphigus vulgaris monoclonal autoantibodies isolated by phage display. J Clin Invest. (2005) 115:888–99. 10.1172/JCI24185 15841178 PMC1070425

[67] Cummins DL Mimouni D Tzu J Owens N Anhalt GJ Meyerle JH. Lichenoid paraneoplastic pemphigus in the absence of detectable antibodies. J Am Acad Dermatol. (2007) 56:153–9. 10.1016/j.jaad.2006.06.007 17097371

[68] Lim JM Kim JH Hashimoto T Kim SC. Lichenoid paraneoplastic pemphigus associated with follicular lymphoma without detectable autoantibodies. Clin Exp Dermatol. (2018) 43:613–5. 10.1111/ced.13563 29761536

[69] Reich K Brinck U Letschert M Blaschke V Dames K Braess J. Graft-versus-host disease-like immunophenotype and apoptotic keratinocyte death in paraneoplastic pemphigus. Br J Dermatol. (1999) 141:739–46. 10.1046/j.1365-2133.1999.03123.x 10583130

[70] Takahashi H Kouno M Nagao K Wada N Hata T Nishimoto S. Desmoglein 3-specific CD4+ T cells induce pemphigus vulgaris and interface dermatitis in mice. J Clin Invest. (2011) 121:3677–88. 10.1172/JCI57379 21821914 PMC3163963

[71] Bennett DD Busick TL. Delayed detection of autoantibodies in paraneoplastic pemphigus. J Am Acad Dermatol. (2007) 57:1094–5. 10.1016/j.jaad.2007.08.019 18021856

[72] Stevens SR Griffiths CE Anhalt GJ Cooper KD. Paraneoplastic pemphigus presenting as a lichen planus pemphigoides-like eruption. Arch Dermatol. (1993) 129:866–9. 10.1001/archderm.1993.01680280054010 8323308

[73] Hoffman MA Qiao X Anhalt GJ. CD8+ T lymphocytes in bronchiolitis obliterans, paraneoplastic pemphigus, and solitary Castleman's disease. N Engl J Med. (2003) 349:407–8. 10.1056/NEJM200307243490421 12878753

[74] Anderson MS Su MA. AIRE expands: new roles in immune tolerance and beyond. Nat Rev Immunol. (2016) 16:247–58. 10.1038/nri.2016.9 26972725 PMC4831132

[75] Barbetakis N Samanidis G Paliouras D Boukovinas I Asteriou C Stergiou E. Paraneoplastic pemphigus regression after thymoma resection. World J Surg Oncol. (2008) 6:83. 10.1186/1477-7819-6-83 18699992 PMC2527607

[76] Shelly S Agmon-Levin N Altman A Shoenfeld Y. Thymoma and autoimmunity. Cell Mol Immunol. (2011) 8:199–202. 10.1038/cmi.2010.74 21317916 PMC4012878

[77] Lee SE Hashimoto T Kim SC. No mucosal involvement in a patient with paraneoplastic pemphigus associated with thymoma and myasthenia gravis. Br J Dermatol. (2008) 159:986–8. 10.1111/j.1365-2133.2008.08763.x 18644017

[78] Strobel P Murumagi A Klein R Luster M Lahti M Krohn K. Deficiency of the autoimmune regulator AIRE in thymomas is insufficient to elicit autoimmune polyendocrinopathy syndrome type 1 (APS-1). J Pathol. (2007) 211:563–71. 10.1002/path.2141 17334980

[79] Marx A Porubsky S Belharazem D Saruhan-Direskeneli G Schalke B Strobel P. Thymoma related myasthenia gravis in humans and potential animal models. Exp Neurol. (2015) 270:55–65. 10.1016/j.expneurol.2015.02.010 25700911

[80] Wada N Nishifuji K Yamada T Kudoh J Shimizu N Matsumoto M. Aire-dependent thymic expression of desmoglein 3, the autoantigen in pemphigus vulgaris, and its role in T-cell tolerance. J Invest Dermatol. (2011) 131:410–7. 10.1038/jid.2010.330 21048786

[81] Wang X Laan M Bichele R Kisand K Scott HS Peterson P. Post-Aire maturation of thymic medullary epithelial cells involves selective expression of keratinocyte-specific autoantigens. Front Immunol. (2012) 3:19. 10.3389/fimmu.2012.00019 22448160 PMC3310317

[82] Finnish-German AC An autoimmune disease, APECED, caused by mutations in a novel gene featuring two PHD-type zinc-finger domains. Nat Genet. (1997) 17:399–403. 10.1038/ng1297-399 9398840

[83] Nagamine K Peterson P Scott HS Kudoh J Minoshima S Heino M. Positional cloning of the APECED gene. Nat Genet. (1997) 17:393–8. 10.1038/ng1297-393 9398839

[84] Kluger N Krohn K Ranki A. Absence of some common organ-specific and non-organ-specific autoimmunity in autoimmune polyendocrinopathy candidiasis ectodermal dystrophy. Endocr Connect. (2013) 2:61–8. 10.1530/EC-12-0074 23781320 PMC3680957

[85] Tsuchisaka A Kaneko S Imaoka K Ota M Kishimoto K Tomaru U. Presence of autoimmune regulator and absence of desmoglein 1 in a thymoma in a patient with pemphigus foliaceus. Br J Dermatol. (2015) 173:268–71. 10.1111/bjd.13617 25523433

[86] Takaba H Morishita Y Tomofuji Y Danks L Nitta T Komatsu N. Fezf2 orchestrates a thymic program of self-antigen expression for immune tolerance. Cell. (2015) 163:975–87. 10.1016/j.cell.2015.10.013 26544942

[87] Fathman CG Lineberry NB. Molecular mechanisms of CD4+ T-cell anergy. Nat Rev Immunol. (2007) 7:599–609. 10.1038/nri2131 17612584

[88] Griffith TS Ferguson TA. Cell death in the maintenance and abrogation of tolerance: the five Ws of dying cells. Immunity. (2011) 35:456–66. 10.1016/j.immuni.2011.08.011 22035838 PMC3205359

[89] Greaves P Gribben JG. The role of B7 family molecules in hematologic malignancy. Blood. (2013) 121:734–44. 10.1182/blood-2012-10-385591 23223433 PMC3563361

[90] Dorfman DM Schultze JL Shahsafaei A Michalak S Gribben JG Freeman GJ. *In vivo* expression of B7-1 and B7-2 by follicular lymphoma cells can prevent induction of T-cell anergy but is insufficient to induce significant T-cell proliferation. Blood. (1997) 90:4297–306. 9373240

[91] Vyth-Dreese FA Boot H Dellemijn TA Majoor DM Oomen LC Laman JD. Localization *in situ* of costimulatory molecules and cytokines in B-cell non-Hodgkin's lymphoma. Immunology. (1998) 94:580–6. 10.1046/j.1365-2567.1998.00550.x 9767448 PMC1364238

[92] Chaperot L Plumas J Jacob MC Bost F Molens JP Sotto JJ. Functional expression of CD80 and CD86 allows immunogenicity of malignant B cells from non-Hodgkin's lymphomas. Exp Hematol. (1999) 27:479–88. 10.1016/S0301-472X(98)00059-9 10089910

[93] Romano C De Fanis U Sellitto A Dalla Mora L Chiurazzi F Giunta R. Effects of preactivated autologous T lymphocytes on CD80, CD86 and CD95 expression by chronic lymphocytic leukemia B cells. Leuk Lymphoma. (2003) 44:1963–71. 10.1080/1042819031000111026 14738151

[94] Van den Hove LE Van Gool SW Vandenberghe P Bakkus M Thielemans K Boogaerts MA CD40 triggering of chronic lymphocytic leukemia B cells results in efficient alloantigen presentation and cytotoxic T lymphocyte induction by up-regulation of CD80 and CD86 costimulatory molecules. Leukemia. (1997) 11:572–80. 10.1038/sj.leu.2400598 9096698

[95] Pasikowska M Walsby E Apollonio B Cuthill K Phillips E Coulter E. Phenotype and immune function of lymph node and peripheral blood CLL cells are linked to transendothelial migration. Blood. (2016) 128:563–73. 10.1182/blood-2016-01-683128 27252234

[96] Maeda Y Nishikawa H Sugiyama D Ha D Hamaguchi M Saito T. Detection of self-reactive CD8(+) T cells with an anergic phenotype in healthy individuals. Science. (2014) 346:1536–40. 10.1126/science.aaa1292 25525252

[97] Johnson DB Sullivan RJ Ott PA Carlino MS Khushalani NI Ye F. Ipilimumab therapy in patients with advanced melanoma and preexisting autoimmune disorders. JAMA Oncol. (2016) 2:234–40. 10.1001/jamaoncol.2015.4368 26633184

[98] Sakaguchi S Vignali DA Rudensky AY Niec RE Waldmann H. The plasticity and stability of regulatory T cells. Nat Rev Immunol. (2013) 13:461–7. 10.1038/nri3464 23681097

[99] Sawant DV Vignali DA. Once a Treg, always a Treg? Immunol Rev. (2014) 259:173–91. 10.1111/imr.12173 24712466 PMC4008876

[100] Haeberle S Wei X Bieber K Goletz S Ludwig RJ Schmidt E. Regulatory T-cell deficiency leads to pathogenic bullous pemphigoid antigen 230 autoantibody and autoimmune bullous disease. J Allergy Clin Immunol. (2018) 142:1831–1842.e1837. 10.1016/j.jaci.2018.04.006 29704595

[101] Muramatsu K Ujiie H Kobayashi I Nishie W Izumi K Ito T. Regulatory T-cell dysfunction induces autoantibodies to bullous pemphigoid antigens in mice and human subjects. J Allergy Clin Immunol. (2018) 142:1818–1830.e1816. 10.1016/j.jaci.2018.03.014 29704593

[102] McGinness JL Bivens MM Greer KE Patterson JW Saulsbury FT. Immune dysregulation, polyendocrinopathy, enteropathy, X-linked syndrome (IPEX) associated with pemphigoid nodularis: a case report and review of the literature. J Am Acad Dermatol. (2006) 55:143–8. 10.1016/j.jaad.2005.08.047 16781310

[103] Kimura A Kishimoto T. IL-6: regulator of Treg/Th17 balance. Eur J Immunol. (2010) 40:1830–5. 10.1002/eji.201040391 20583029

[104] Bettelli E Carrier Y Gao W Korn T Strom TB Oukka M. Reciprocal developmental pathways for the generation of pathogenic effector TH17 and regulatory T cells. Nature. (2006) 441:235–8. 10.1038/nature04753 16648838

[105] Korn T Bettelli E Gao W Awasthi A Jager A Strom TB. IL-21 initiates an alternative pathway to induce proinflammatory T(H)17 cells. Nature. (2007) 448:484–7. 10.1038/nature05970 17581588 PMC3805028

[106] Fujimoto M Serada S Mihara M Uchiyama Y Yoshida H Koike N. Interleukin-6 blockade suppresses autoimmune arthritis in mice by the inhibition of inflammatory Th17 responses. Arthritis Rheum. (2008) 58:3710–9. 10.1002/art.24126 19035481

[107] Pasare C Medzhitov R. Toll pathway-dependent blockade of CD4+CD25+ T cell-mediated suppression by dendritic cells. Science. (2003) 299:1033–6. 10.1126/science.1078231 12532024

[108] Vinuesa CG Linterman MA Yu D MacLennan IC Follicular helper T cells. Annu Rev Immunol. (2016) 34:335–68. 10.1146/annurev-immunol-041015-055605 26907215

[109] Nousari HC Kimyai-Asadi A Anhalt GJ. Elevated serum levels of interleukin-6 in paraneoplastic pemphigus. J Invest Dermatol. (1999) 112:396–8. 10.1046/j.1523-1747.1999.00520.x 10084324

[110] Lee SH Hong WJ Kim SC. Analysis of serum cytokine profile in pemphigus. Ann Dermatol. (2017) 29:438–45. 10.5021/ad.2017.29.4.438 28761292 PMC5500709

[111] Fajgenbaum DC. Novel insights and therapeutic approaches in idiopathic multicentric Castleman disease. Blood. (2018) 132:2323–30. 10.1182/asheducation-2018.1.318 30487129 PMC6265649

[112] Chen YJ Chang JT Lee L Wang HM Liao CT Chiu CC. DSG3 is overexpressed in head neck cancer and is a potential molecular target for inhibition of oncogenesis. Oncogene. (2007) 26:467–76. 10.1038/sj.onc.1209802 16878157

[113] Parikka M Kainulainen T Tasanen K Vaananen A Bruckner-Tuderman L Salo T. Alterations of collagen XVII expression during transformation of oral epithelium to dysplasia and carcinoma. J Histochem Cytochem. (2003) 51:921–9. 10.1177/002215540305100707 12810842

[114] Yamada T Endo R Tsukagoshi K Fujita S Honda K Kinoshita M. Aberrant expression of a hemidesmosomal protein, bullous pemphigoid antigen 2, in human squamous cell carcinoma. Lab Invest. (1996) 75:589–600. 8874389

[115] Herold-Mende C Kartenbeck J Tomakidi P Bosch FX. Metastatic growth of squamous cell carcinomas is correlated with upregulation and redistribution of hemidesmosomal components. Cell Tissue Res. (2001) 306:399–408. 10.1007/s004410100462 11735040

[116] Bowen GM Peters NT Fivenson DP Su LD Nousari HC Anhalt GJ. Lichenoid dermatitis in paraneoplastic pemphigus: a pathogenic trigger of epitope spreading? Arch Dermatol. (2000) 136:652–6. 10.1001/archderm.136.5.652 10815859

